# Competitive pressures affect sexual signal complexity in *Kurixalus odontotarsus*: insights into the evolution of compound calls

**DOI:** 10.1242/bio.028928

**Published:** 2017-11-24

**Authors:** Bicheng Zhu, Jichao Wang, Zhixin Sun, Yue Yang, Tongliang Wang, Steven E. Brauth, Yezhong Tang, Jianguo Cui

**Affiliations:** 1 Chengdu Institute of Biology, Chinese Academy of Sciences, Chengdu 610041, Sichuan, China; 2 University of Chinese Academy of Sciences, Beijing 100049, China; 3 Department of Biology, Hainan Normal University, Haikou 571158, Hainan, China; 4 Department of Psychology, University of Maryland, College Park, MD 20742, USA

**Keywords:** Sexual selection, Call plasticity, Signal evolution, Male-male competition, *Kurixalus odontotarsus*

## Abstract

Male-male vocal competition in anuran species is critical for mating success; however, it is also energetically demanding and highly time-consuming. Thus, we hypothesized that males may change signal elaboration in response to competition in real time. Male serrate-legged small treefrogs (*Kurixalus odontotarsus*) produce compound calls that contain two kinds of notes, harmonic sounds called ‘A notes’ and short broadband sounds called ‘B notes’. Using male evoked vocal response experiments, we found that competition influences the temporal structure and complexity of vocal signals produced by males. Males produce calls with a higher ratio of notes:call, and more compound calls including more A notes but fewer B notes with contest escalation. In doing so, males minimize the energy costs and maximize the benefits of competition when the level of competition is high. This means that the evolution of sexual signal complexity in frogs may be susceptible to selection for plasticity related to adjusting performance to the pressures of competition, and supports the idea that more complex social contexts can lead to greater vocal complexity.

## Abstract (precis)

**Summary:** Competitive pressure influences the temporal structure and complexity of vocal signals. The evolution of sexual signal complexity may be susceptible to selection for plasticity related to adjusting performance to the level of competition.

## INTRODUCTION

The signal design of animal calls is thought to be shaped by many factors, including social contexts (Hauser, 1993; Brenowitz and Rose, 1999; Cui et al., 2010; Freeberg et al., 2012; Gustison et al., 2012; Krams et al., 2012; Maciej et al., 2013). For example, male vocal competition in music frogs (*Babina daunchina*) is strongly affected by social contexts and males allocate competitive efforts depending on both the perceived sexual attractiveness of rivals and the time available for calling (Fang et al., 2014). Generally, the variation in graded calling signals is correlated with variation in competition, and the variation of population density along with different temporal and spatial cues will influence the dynamics of the contest (Wells, 1989; Jirotkul, 1999; Lange and Leimar, 2003). Dense choruses make communication more difficult.

As animal contests escalate, variation in call signaling behaviors can reveal how vocal strategy may be adjusted based on temporal competition (Dyson et al., 2013; Bee et al., 2016). For example, when interacting acoustically at close range with other males, males of many species switch from the production of advertisement calls to the production of aggressive calls (Wells and Schwartz, 1984; Wells, 1988; Grafe, 1995; Jehle and Arak, 1998). Male gray treefrogs (*Hyla versicolor*) increase calling rate but decrease call duration during manipulations of chorus size up to eight callers, and lower their aggressive call frequencies in more escalated contests. These findings suggest that escalated contests may promote the generation of graded aggressive signals (Schwartz et al., 2002; Reichert and Gerhardt, 2013). These findings also strengthen the idea that competitive pressures influence anuran calling behavior and promote the evolution of sexual signals. Nevertheless, despite an abundance of research on acoustic communication and call plasticity in anurans, relatively little is known about the fine-scale adjustments in the internal structure of calls produced in response to contest escalation.

Vocal competition in anuran species is critical for mating success; however, it is also highly energetically demanding and time consuming, likely to increase predation risks (Gerhardt and Huber, 2002; Contreras-Garduno et al., 2007; Bradbury and Vehrencamp, 2011). The complex signals produced in highly escalated competitions entail high energy expenditure and increase the risk of predator detection (Hartbauer et al., 2012). Therefore, dynamic male competitive strategies sensitive to cues in the competitive environment appear to have evolved in frogs, which serve to maximize the likelihood that individual males defeat rivals and attract potential mates in the lek or chorus. The present study investigated this problem in the serrate-legged small treefrog, *Kurixalus odontotarsus* – a tropical species in which males produce calls with graded complexity during the breeding season (Fig. S1). The hypothesis tested here questions if *K. odontotarsus* males can make adjustments in the temporal structure and complexity of vocal signals which would allow them to minimize the energy costs and maximize the benefits of competition when the level of competition is high.

Males can distinguish nearby rivals and estimate the level of competition through vocal signals in the lek (Wilczynski and Brenowitz, 1988; Brenowitz, 1989; Gerhardt et al., 1989). For this reason we used recordings of calls produced in natural groups of different densities to simulate varying levels of competition in *K. odontotarsus*. Most of the calls of *K. odontotarsus* consist of two note types called ‘note A’ and ‘note B’. Males typically produce three kinds of calls with these notes: A-note calls, B-note calls and compound calls containing both kinds of notes (Zhu et al., 2017a). Previous research has shown that note A acts as advertisement calls while note B acts as an aggressive call to suppress competitors' advertisement calls. Compound calls produced by male *K. odontotarsus* contain both kinds of notes, insofar as these calls both attract females and suppress competitors (Zhu et al., 2017b). The existence of several call types in *K. odontotarsus* provides an excellent opportunity to study the effect of competition on signaling behavior. In the present study, we used male evoked vocal response experiments in *K. odontotarsus* to test the hypotheses that males may adjust the temporal structure and complexity of vocal signals based on competitive pressures. This hypothesis is consistent with the idea that high levels of competition favor the evolution of sexual signal complexity.

## RESULTS

### Male evoked vocal responses

A total of 58 males were utilized in the playback tests, and 1566 min of calling responses were recorded. We analyzed the total number of notes, notes:call, maximum number of notes in advertisement calls, the number of compound calls, and the numbers of note A and note B in these compound calls during male evoked vocal responses.

**Fig. 1.**
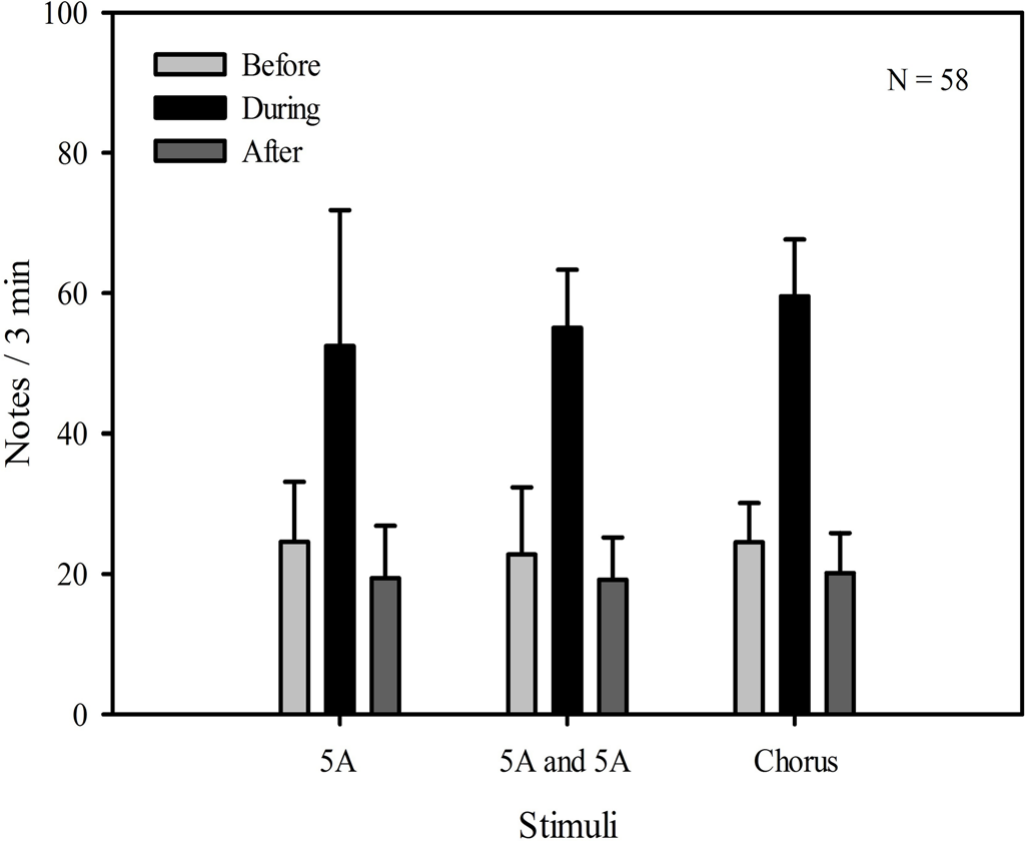
**Male *K. odontotarsus* evoked vocal responses: the total number of notes produced in response to the three stimulus types.** Two-way repeated measures ANOVA and Holm-Sidak method. Data are expressed as mean±s.d.

There were significant differences in the total number of notes produced during male evoked vocal responses between playback time (*F*_2, 57_=64.801, *P*<0.001, two-way repeated measures ANOVA), but not stimuli (*F*_2, 57_=0.448, *P*=0.640). There were no statistically significant interaction effects between playback time and stimuli (playback time×sound interaction effect, *F*_2, 2_=0.497, *P*=0.738). For all stimuli, male calling responses during playbacks differed significantly from those produced before (differential of means=31.674, t=9.160, *P*<0.001, Holm-Sidak method) and after (differential of means =36.080, t=10.434, *P*<0.001; Fig. 1, Table 1). Male calling responses before playbacks were similar and not significantly different from those produced after (differential of means =4.406, t=1.274, *P*=0.206; Fig. 1, Table 1).

**Table 1.**
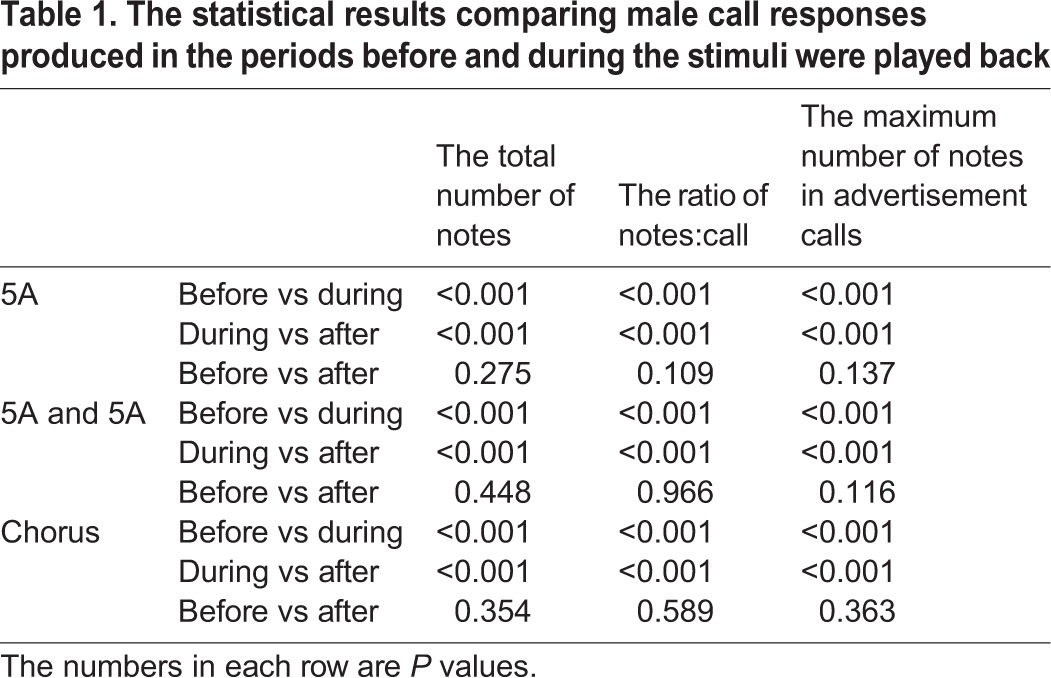
**The statistical results comparing male call responses produced in the periods before and during the stimuli were played back**

**Fig. 2.**
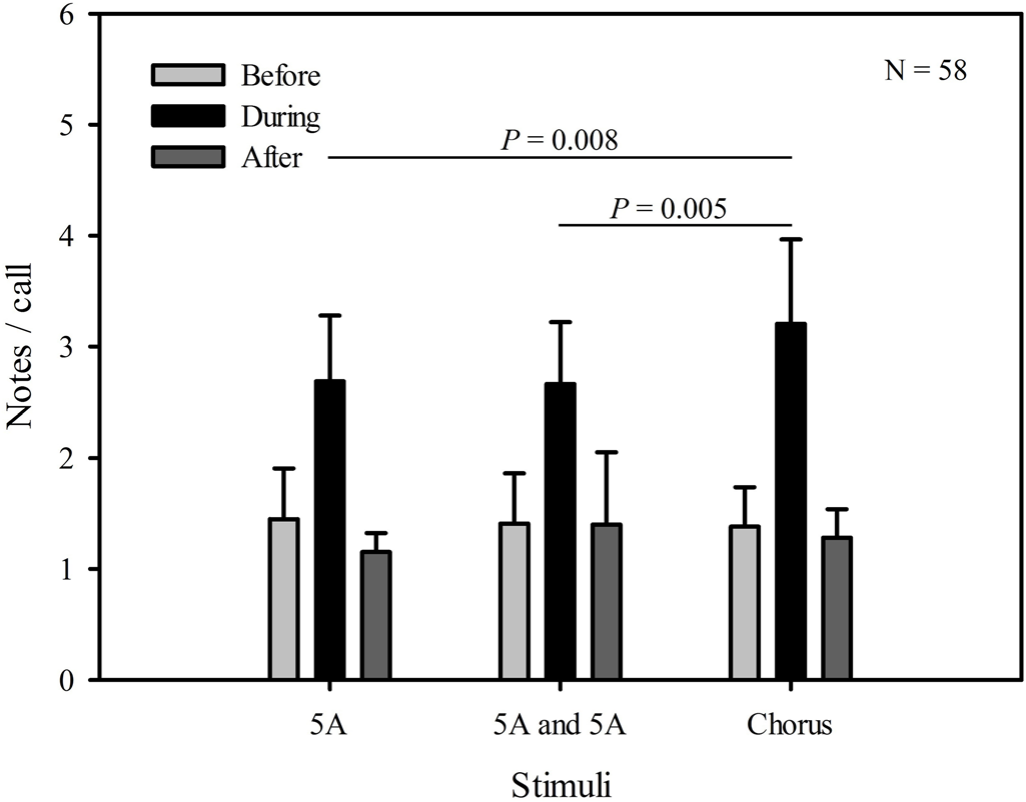
**Male *K. odontotarsus* evoked vocal responses: the ratio of notes/call in response to the three stimulus types**. Two-way repeated measures ANOVA and Holm-Sidak method. Data are expressed as mean±s.d.

We based our measures of call complexity in *K. odontotarsus* on previous studies of signal complexity in túngara frogs (Bernal et al., 2009; Akre et al., 2011). One simple measure of signal complexity is the ratio of notes:call. In our data there are significant differences in the ratio of notes:call between playback time (*F*_2, 57_=103.531, *P*<0.001), but not stimuli (*F*_2, 57_=1.083, *P*=0.343). There was a statistically significant interaction effect between playback time and stimuli (playback time×sound interaction effect, *F*_2,2_=2.804, *P*=0.027). Using a multiple comparison procedure, we then found no significant difference between the ratio of notes:call during the single five A note stimulus group playback period and during the two five A notes stimulus group playback period (differential of means=0.0235, t=0.122, *P*=0.903). However, males produced calls with a higher ratio of notes:call during the period the chorus stimulus group were played back than during both the single five A note stimulus group playback period (differential of means=0.518, t=2.685, *P*=0.008), and during the two five A notes stimulus group playback period (differential of means=0.541, t=2.807, *P*=0.005, Fig. 2, Table 1). For all stimuli, the ratio of notes:call before playbacks were similar and not significantly different from those produced after (differential of means=0.135, t=1.110, *P*=0.270, Fig. 2, Table 1).

**Fig. 3.**
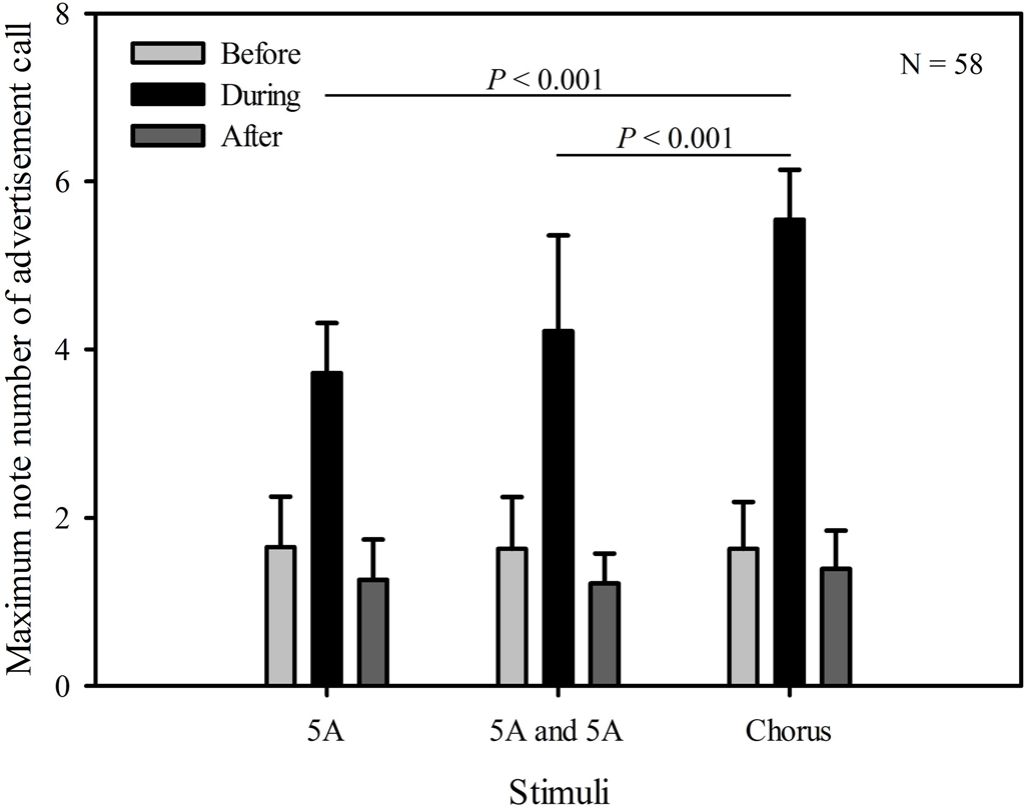
**Male *K. odontotarsus* evoked vocal responses: the maximum number of notes in advertisement calls in response to the three stimulus types**. Two-way repeated measures ANOVA and Holm-Sidak method. Data are expressed as mean±s.d.

The maximum number of notes in advertisement calls, to some degree, likely reflects the complexity of a vocal signal as well. After analyzing the maximum number of notes in advertisement calls produced in response to the three different stimuli, we found that there were significant differences in the maximum note number of advertisement calls between playback time (*F*_2,57_=214.673, *P*<0.001), and stimuli (*F*_2,57_=7.951, *P*<0.001). There was a statistically significant interaction effect between playback time and stimuli (playback time×sound interaction effect, *F*_2,2_=9.254, *P*<0.001). Then, using a multiple comparison procedure, we found no significant difference between the maximum number of notes in advertisement calls during the period the single five A note stimulus group were played back and during the period the two five-A-notes stimulus group were played back (differential of means=0.500, t=1.906, *P*=0.058; Fig. 3, Table 1); although males produced advertisement calls with a greater maximum note number during the period the chorus stimulus group were played back than during both the single five A note stimulus group playback period (differential of means=1.826, t=6.961, *P*<0.001), and during the two five-A-notes stimulus group playback period (differential of means=1.326, t=5.055, *P*<0.001; Fig. 3, Table 1). For all stimuli, the maximum note number of advertisement calls before playbacks were similar and not significantly different from those produced after (differential of means=0.348, t=2.011, *P*=0.056; Fig. 3, Table 1).

### Evoked compound calls

Another important measure that has been frequently used in studies of signal complexity is note types. Evoked compound calls contain two different note types, and therefore can be taken to reflect greater call complexity. There were 56 evoked compound calls in 1566 min of recordings. There were 27 evoked compound calls produced in response to the chorus stimulus, 15 evoked compound calls produced in response to the single five A note stimulus, and 14 evoked compound calls produced in response to the two five-A-notes stimulus. All compound calls were produced only during the period the stimuli were played back; thus no playback time effect was computed for male evoked compound calls. One-way repeated measures ANOVA was used to evaluate the differences among the numbers of note A and note B in compound calls during male evoked vocal responses for the three stimulus groups.

**Fig. 4.**
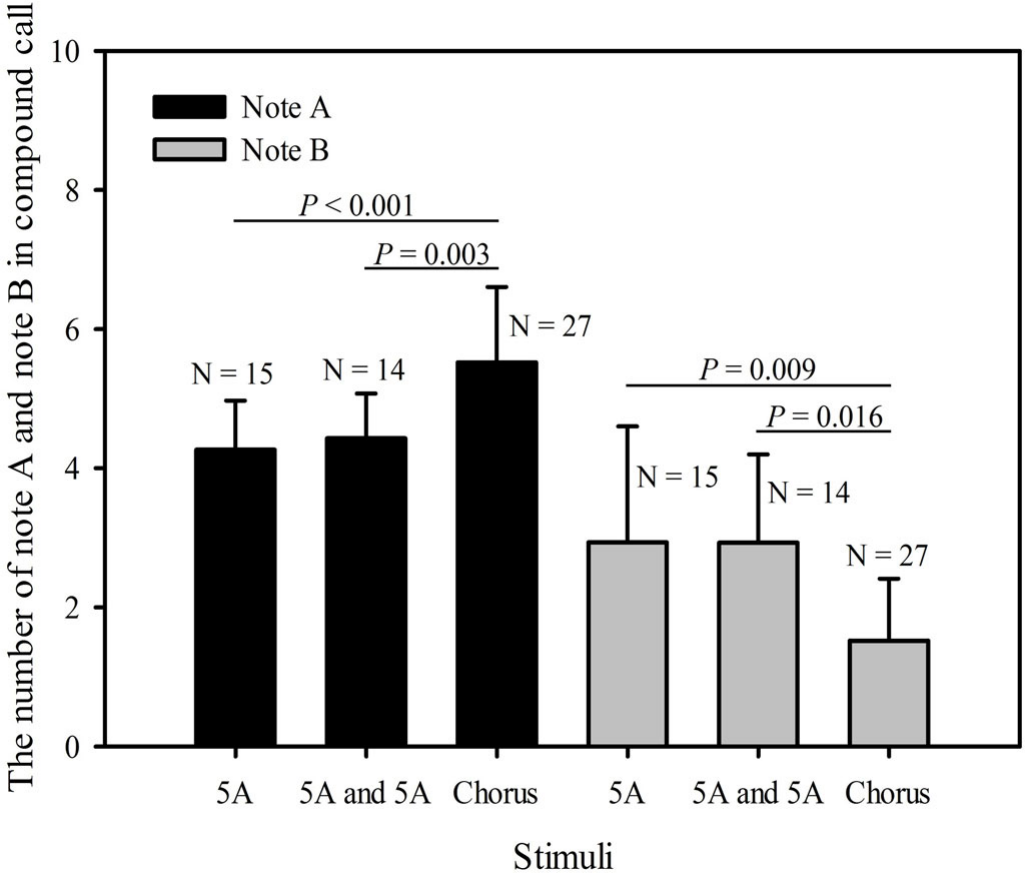
**Male *K. odontotarsus* evoked vocal responses: the numbers of A notes and B notes in compound calls in response to the three stimulus types**. One-way repeated measures ANOVA and Holm-Sidak method. Data are expressed as mean±s.d.

There was no significant differences among the three different stimuli groups in the total note number of compound calls (single five A note: 7.2±1.93 notes, two five-A-notes: 7.4±1.50 notes, chorus: 7.04±1.34 notes; *F*_2_=0.107, *P*=0.899, one-way repeated measures ANOVA). However, the numbers of both A notes and B notes in compound calls differed significantly among the three different stimulus groups (Note A: *F*_2_=8.287, *P*=0.002; Note B: *F*_2_=4.919, *P*=0.015). A multiple comparison procedure revealed that the number of A notes (5.52±1.12) in compound calls produced in response to chorus playbacks was significantly greater than both the number of A notes in compound calls produced in response to the single five A note (4.27±0.70) stimulus (differential of means=1.133, t=3.751, *P*<0.001, Holm-Sidak method; Fig. 4, Table 2) and to the number of A notes in compound calls produced in response to the two five-A-notes (4.43±0.65) stimulus (differential of means=0.995, t=3.209, *P*=0.003; Fig. 4, Table 2). Furthermore, the number of B notes (1.51±0.89) in compound calls produced in response to the chorus playbacks was significantly less than those produced in response to both the single five A note (2.93±1.78) stimulus (differential of means=1.333, t=2.830, *P*=0.009; Fig. 4, Table 2) and the two five-A-notes (2.92±1.27) stimulus (differential of means=1.238, t=2.560, *P*=0.016; Fig. 4). There were no significant differences in the number of A notes in compound calls produced in response to the single five A note stimulus and the two five-A-notes stimulus group (differential of means=0.138, t=0.373, *P*=0.712; Fig. 4) and the number of B notes (differential of means=0.0952, t=0.165, *P*=0.870; Fig. 4, Table 2).

**Table 2.**
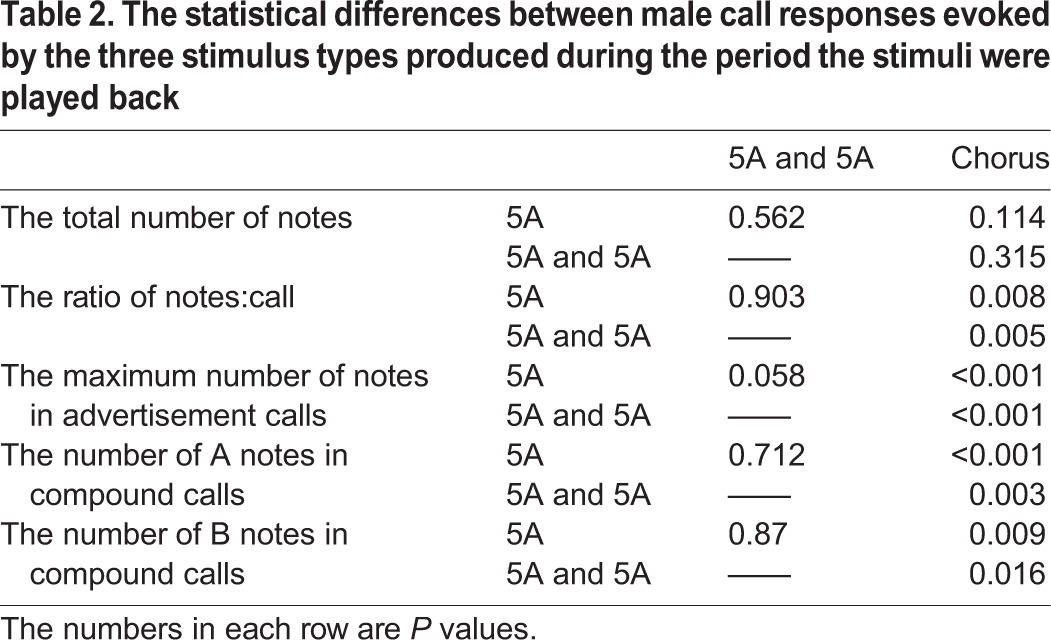
**The statistical differences between male call responses evoked by the three stimulus types produced during the period the stimuli were played back**

## DISCUSSION

Our results show that male *K. odontotarsus* produced more complex calls (higher ratio of notes:call, more compound calls) in response to the chorus stimulus (representing a high level of competition) than in response to either the single or two five-note-A call stimuli (representing low or moderate levels of competition). These results suggest that males can adjust calling behavior with contest escalation, and are consistent with the idea that competitive pressures may enhance the evolution of sexual signal complexity in frogs (West-Eberhard, 1983; Andersson, 1994). In addition, the results of the present study reveal no significant differences in the overall numbers of notes produced by subjects in response to the three different stimuli, consistent with the idea that changes in calling associated with differing levels of competition are due to changes in the temporal structure (or complexity) of the calls rather than changes in the overall calling output. For all stimuli, the total number of notes, the ratio of notes:call and the maximum number of notes in advertisement calls before playbacks were similar and not significantly different from those produced after, consistent with the idea that male evoked vocal responses produced before and after the stimuli were broadcast reflecting baseline levels. Thus, after the stimuli were broadcast, males resumed spontaneous calling.

The present study reveals that competition may be an impetus for promoting selection for signal exaggeration. In some species, males produce a single type of sexual signal to attract females and compete with rivals, while others have evolved very complex and variable sexual signals (Narins et al., 2000; Christensen-Dalsgaard et al., 2002; Cui et al., 2016; Zhu et al., 2016). The Madagascar bright-eyed frog (*Boophis madagascariensis*) has an extraordinarily varied vocal repertoire of at least 28 different call types (Narins et al., 2000). Mate choice is always accompanied by competitive pressures. Our study further supports the idea that competitive pressure likely enhances the evolution of sexual signal complexity in anurans.

The size and densities of male aggregations vary among natural treefrog choruses as males enter amplexus or cease calling. Theoretically, plastic changes in calling behavior should be favored when population density and mean male crowding are high and intrasexual competition is fierce (Schuster and Wade, 2003; Oliveira et al., 2008). Male *Xenopus laevis* can adjust call duration based on the presence of potential mates in order to avoid wasting ‘unnecessary’ energy (Xu et al., 2012). Males of some species (e.g. *Rana catesbeiana*) lower the fundamental frequency of their calls in response to those of neighboring males in order to be more competitive, because rivals are more likely to retreat from the low-pitched calls of large males (Bee and Bowling, 2002). Driven by different levels of competition, *K. odontotarsus* males adjust calling behavior with contest escalation and produce more compound calls under high levels of competition. These compound calls can simultaneously attract females and suppress rivals. Therefore, males producing more compound calls when competition is great can benefit from both female choice and male aggression.

Males compete directly for access to resources or females and indirectly for female mate choice in most animal species. Competition between males for potential mates is highly intense in *K. odontotarsus*. Intense male competitive callings are typically accompanied by high energy expenditure (Hartbauer et al., 2012). *K. odontotarsus* males produce compound calls with more A notes under high levels of competition in comparison with those produced under lower levels of competition. These results are consistent with previous results of female phonotaxis experiments, showing that females prefer calls containing more A notes. It is quite interesting that males produce compound calls with fewer B notes under high levels of competition than are produced under lower levels of competition. This is because the suppressing effects of note B are greatest for compound calls containing two B notes and are absent if males produce five B notes in a compound call. Furthermore, while female *K. odontotarsus* prefer compound calls to simpler calls with only A notes, female preference does not increase when the number of B notes in compound calls increases from two to five (Zhu et al., 2017b). Finally, producing compound calls with fewer B notes is less energy intensive under high levels of competition than under lower levels of competition. This explains why 85.6% of compound calls produced in natural contexts contain only one or two B notes, and why it is unusual for males to produce five B notes in a compound call. The ability to make such fine adjustments in the inter-note structure of compound calls allows males to balance the costs of vocal signaling with the benefits of increasing attractiveness to females or effectively suppressing rivals on the basis of the competitive environment.

## MATERIALS AND METHODS

### Acoustic stimuli

**Fig. 5.**
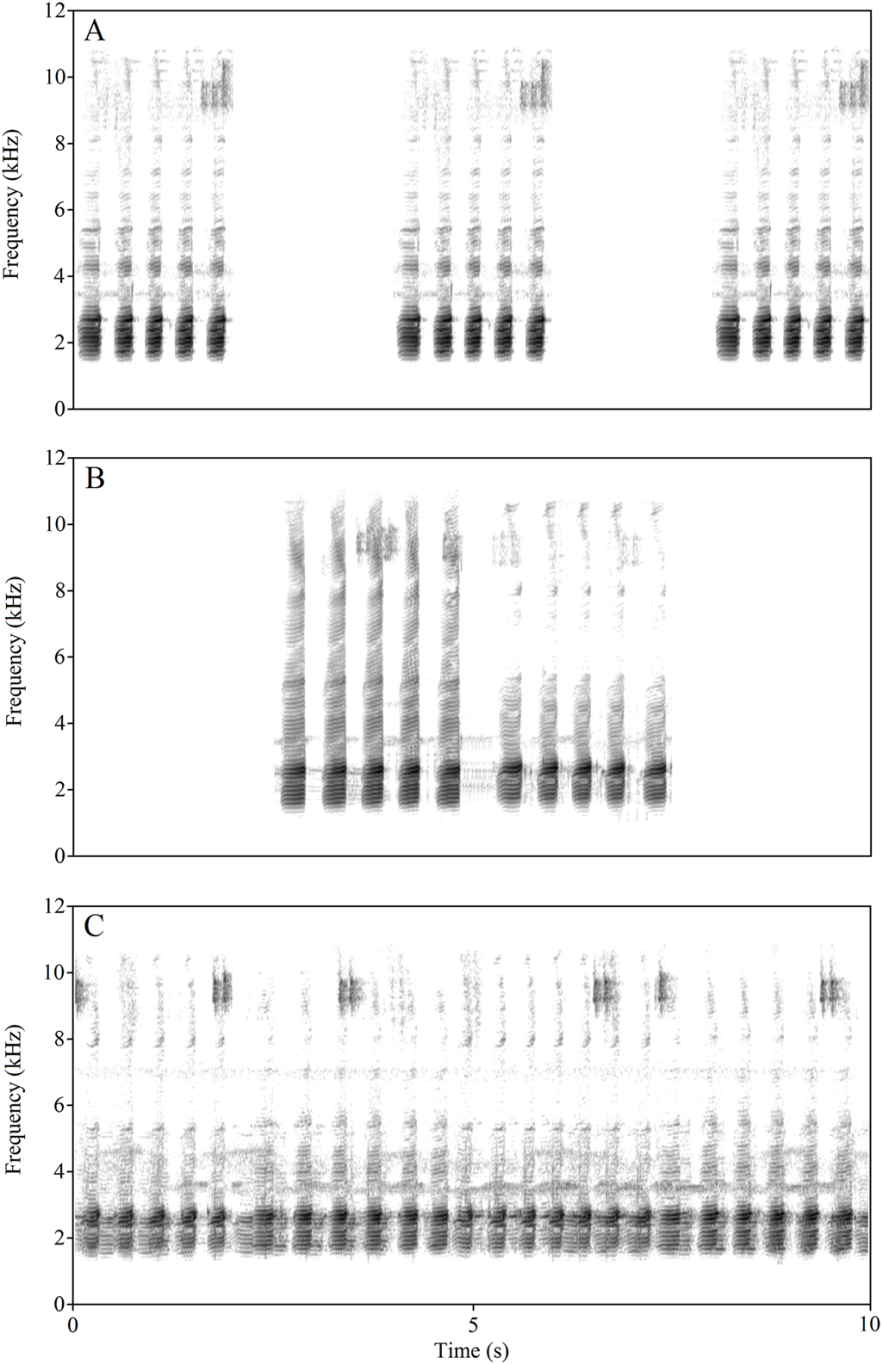
**The spectrograms of the three stimuli.** A single call consisting of five A notes representing low a level of competition (A), a call consisting of one five-A-note call followed by another representing a moderate level of competition (B), a chorus consisting of calls representing a high level of competition (C). The FFT (fast Fourier transform) frame is 1024.

To evaluate the influence of competition on male signal production, three stimulus types were constructed for male playback tests as follows: calls consisting of five A notes (2 s, representing a low level of competition, Fig. 5A); calls consisting of one five-A-notes followed by another five-A-notes call (5 s, representing a moderate level of competition, Fig. 5B); a natural chorus without B notes (10 s, representing a high level of competition, Fig. 5C). Each stimulus was played with 2-s (stimulus ‘5A’), 5-s (stimulus ‘5A and 5A’) and 10-s (stimulus ‘chorus’) inter-stimulus intervals respectively, to ensure the same total stimulation duration (90 s) for each. Note that the stimulus consisting of one five-A-notes call followed by another is not a simple repetition of the same five-A-notes call, because each five-A-notes call was recorded from a different male. Furthermore, the inter-call interval between the first and second five-A-notes call was 0.43±0.05 s, far shorter than the inter-stimulus interval of the stimulus call consisting of only one five-A-notes call (2 s), but closer to the inter-note interval of the five-A-notes call (0.22±0.04 s). All stimuli were constructed using natural calls. To avoid pseudoreplication effects, five exemplars of each call type, derived from five different calling males were collected (McGregor et al., 1992). To minimize bias, observers were blind to the experimental conditions in use during recording and analysis of all male response data.

### Male evoked vocal response experiments

Male evoked vocal response experiments were conducted in the Mt. Diaoluo National Nature Reserve in Hainan, China (18.44°N, 109.52°E, elevation of 933 m a.s.l.) from July to August, 2014. Given that male serrate-legged small treefrogs failed to respond in sound-attenuating chambers, calling males were captured at their breeding sites and brought to a field test site where no males were calling nearby, but where the environment was similar to their breeding sites. We tested males in evoked vocal response experiments using three stimuli: (1) 5A, (2) 5A and 5A, (3) chorus. To avoid order effects, the stimuli were presented to males in a randomized sequence, between 20:00 h to 24:00 h [temperature: 21.3±0.35°C; relative humidity: 93.5±3.12% (mean±s.d.)]. Each male subject was placed on the top of shrubbery. In a previous study we demonstrated that male frogs compete vocally on the basis of the temporal sequence of rival calls and do not distinguish between two calls played from one speaker and two calls played from two spatially separated speakers (Jiang et al., 2015). Thus we used only one portable field speaker (SME-AFS, Saul Mineroff Electronics, Elmont, NY, USA), which was placed 1 m from the shrubbery (the approximate distance of two nearby males in nature) to broadcast the stimuli. The response calls of males before, during and after playback were recorded for three minutes using an Aigo R5518 recorder with an internal microphone (Aigo Digital Technology Co. Ltd., Beijing). The ‘fast’ root-mean-square amplitude of the broadcast test stimuli at the release position was 80 dB SPL (re 20 μPa, A-weighted, the approximate SPL of a natural call at the same distance). The sound pressure levels (SPLs) of each test call were measured with a sound level meter (AWA 6291, Hangzhou Aihua Instruments Co., Hangzhou, China). The frogs were returned that night to their original habitat after the tests were completed. Prior to being returned to their original habitat, the subjects were given a unique toe-clip number to avoid being retested.

### Analysis and statistics

The sonograms of calls were generated using free PRAAT software (Boersma, 2002). Data were statistically analyzed and figures created using Sigmaplot 11.0 software (Systat Software Inc., Chicago, USA). The effects of stimulus type and playback time (before, during and after playback) on the total number of notes, notes/call, maximum note number of advertisement call during male evoked vocal responses were analyzed using two-way repeated measures ANOVA (two factor repetition). All compound calls were produced only during the period the stimuli were played back. Thus no playback time effect for male evoked compound calls was obtained. One-way repeated measures ANOVA was used to evaluate the differences among the numbers of note A and note B in compound calls during male evoked vocal responses for the three stimulus groups. If a statistically significant difference was found, a multiple comparison procedure (Holm-Sidak method) was used to isolate the group or groups that differed from the others. Prior to statistical analyses, all data were examined for assumptions of normality and homogeneity of variance, using the Shapiro-Wilk and Levene tests, respectively. Data are expressed as mean±s.d., and *P*<0.05 was considered to be statistically significant.

### Ethics note

All applicable international, national, and/or institutional guidelines for the care and use of animals were followed. All procedures performed in studies involving animals were approved by the Animal Care and Use Committee of Chengdu Institute of Biology, CAS (CIB2014031008). This work was conducted with the permission of the Management Office of the Mt. Diaoluo Nature Reserve.

## Supplementary Material

Supplementary information
