# Supplementary figures and images for: Competitive pressures affect sexual signal complexity in Kurixalus odontotarsus: insights into the evolution of compound calls
Source: Biol Open. 2017 Nov 24;6(12):1913–8. doi: 10.1242/bio.028928 (PMC5769655; doi:10.1242/bio.028928)

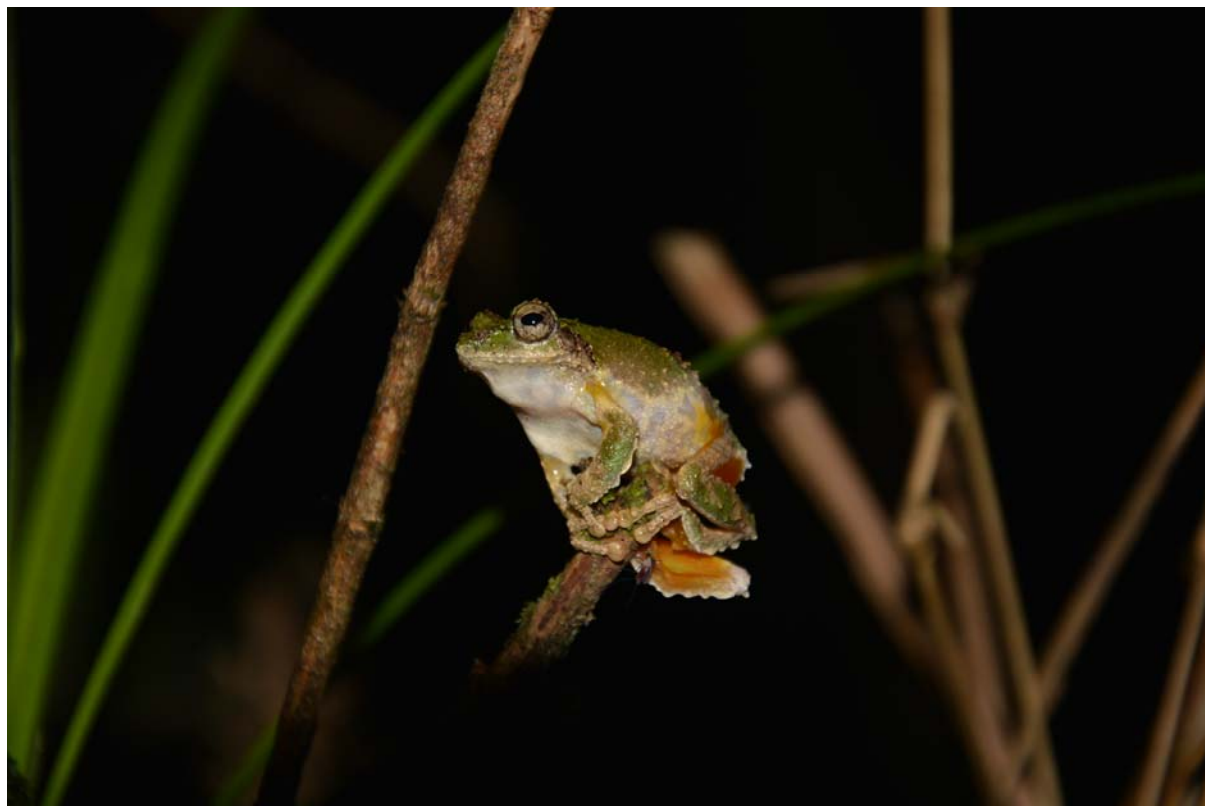

**Fig. S1** *Kurixalus odontotarsus* (male).

Supplement: Supplementary information [file biolopen-6-028928-s1.pdf]
